# Supplementary material for: Rapid and Sensitive Detection of Verticillium dahliae from Soil Using LAMP-CRISPR/Cas12a Technology
Source: Int J Mol Sci. 2024 May 10;25(10):5185. doi: 10.3390/ijms25105185 (PMC11120695; doi:10.3390/ijms25105185)
Supplement: Supplementary file 1 [file ijms-25-05185-s001.zip › ijms-2988758-supplementary.pdf]

Table S1. Sequences of LAMP primers and crRNA.

Primer/crRNA name	sequences（5′→3′）
PCR-F	ATGAAACGTAAGGTAGGTAGC
PCR-R	CCAGAGCAGCGCCTCATTCGG
VD-FIP	TCTCCGTGGATGTTCTCGGGAATAATGGCTGCCGTGACTGTC
VD-BIP	TAGGGACGCAACAATGAGCTGTGCACGGCGCCAAAGTTC
VD-F3	AGCGGAAGGGGCACTAG
VD-B3	CAAAGACCACGACCATAGGC
VD-LF	ACGATTGGCAGTCACGGTT
VD-LB	TTGACGGCTTTACCACAGTCT
crRNA	UAAUUUCUACUAAGUGUAGAUCCACAGUCUUCUCGGCCAAGU
ssDNA-1	(FAM)CCACGGGAGGAATACCAACCCAGTG(BHQ1)
ssDNA-2	(FAM)TTATTATT(Biotin)

Table S2. Strains of *Verticillium dahliae* Used in Research.

Strain	Species	Original location
Vd130	*V. dahliae*	Urumqi,Xinjiang,China
Vd156	*V. dahliae*	Urumqi,Xinjiang,China
Vd179	*V. dahliae*	Urumqi,Xinjiang,China
Vd218	*V. dahliae*	Urumqi,Xinjiang,China
Vd233	*V. dahliae*	Urumqi,Xinjiang,China

Strain	Species	Original location
Vd250	*V. dahliae*	Urumqi,Xinjiang,China
Vd279	*V. dahliae*	Urumqi,Xinjiang,China
Vd300	*V. dahliae*	Urumqi,Xinjiang,China
Vd315	*V.dahliae*	Urumqi,Xinjiang,China
Vd450	*V. dahliae*	Urumqi,Xinjiang,China
Vd690	*V.dahliae*	Urumqi,Xinjiang,China
Vd695	*V.dahliae*	Urumqi,Xinjiang,China
Vd701	*V. dahliae*	Urumqi,Xinjiang,China
Vd722	*V. dahliae*	Urumqi,Xinjiang,China
Vd748	*V. dahliae*	Nanjing,Jiangsu,China
Vd755	*V. dahliae*	Nanjing,Jiangsu,China

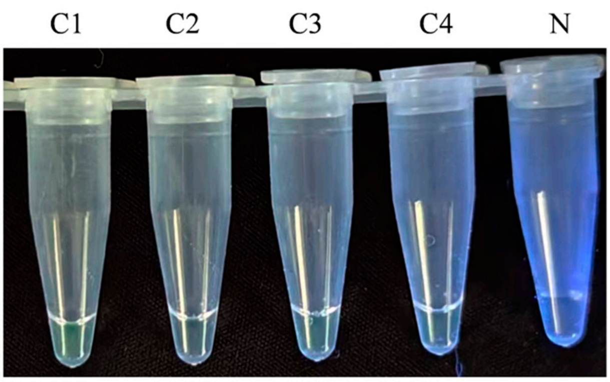

Figure S1. LAMP-CRISPR/Cas12a fluorescence visualization system detects natural soil diseases. C1-C4: Natural soil samples carrying *V. dahliae* from Xinjiang, China; N: Negative.

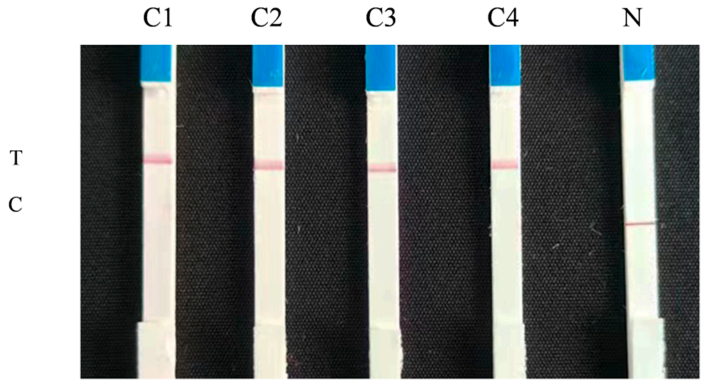

Figure S2. LAMP-CRISPR/Cas12a on-spot system detects natural soil diseases. C1-C4: Natural soil samples carrying *V. dahliae* from Xinjiang, China; N: Negative.
